# A wearable microwave detector for diagnosing thoracic injuries-test on a porcine pneumothorax model

**DOI:** 10.1186/1757-7241-23-S2-A20

**Published:** 2015-09-11

**Authors:** Nils Petter Oveland, Ruben Buendia, Bengt Arne Sjöqvist, Marianne Oropeza-Moe, Nina Gjerde Andersen, Andreas Fhager, Mikael Persson, Mikael Elam, Stefan Candefjord

**Affiliations:** 1 Department of Health Studies, Network for Medical Sciences, University of Stavanger, Stavanger, Norway; 2 Department of Anesthesiology and Intensive Care, Stavanger University Hospital, Stavanger, Norway; 3 Department of Research and Development, Norwegian Air Ambulance Foundation, Droebak, Norway; 4 Chalmers University of Technology, Gothenburg, Sweden; 5 MedTech West, Gothenburg, Sweden; 6 SAFER Vehicle and Traffic Safety Centre at Chalmers University of Technology, Gothenburg, Sweden; 7 Department of Production Animal Clinical Sciences, Faculty of Vetbio, Norwegian University of Life Sciences, Sandnes, Norway; 8 Sahlgrenska University Hospital, Gothenburg, Sweden

## Background

In the prehospital setting, a point-of-care diagnostic test is needed to diagnose pneumothorax (PTX) and monitor its progression to prevent unnecessary patient morbidity and mortality. Ultrasonography is more sensitive than supine chest x-ray for diagnosing PTX, but the accuracy depends on the experience of the operator. Therefore, a non-operator dependent instrument would be valuable for detection and continuous monitoring of an evolving PTX.

## Study objective

To evaluate the potential of a new microwave technology for diagnosing PTX.

## Methods

An experimental PTX model was set up in two anesthetized pigs. A belt with eight microwave antennas was strapped around the pig's chest. Air was insufflated into a catheter in the right pleural space in twelve incremental steps (PTX volumes: 50, 100, 150, 200, 250, 300, 400, 500, 750, 1000, 1500, 2000 mL). Each injection was followed by a measurement with the microwave detector. A computer-based classification algorithm was used to distinguish between the measurements using a leave-one-out approach (i.e. the sample to be classified was not included in the training data matrix), where each PTX volume was treated as an individual class.

## Results

The microwave belt was able to differentiate between normal lungs and PTX in both animals with an overall diagnostic accuracy of 100 % (i.e. a sensitivity and specificity of 100%). Furthermore, the classification accuracy for predicting the size of PTX was 100% and 98% for each pig, respectively.

## Conclusion

The microwave technology proved promising in diagnosing and predicting size of PTX ranging from 50 mL to 2000 mL. This within-model experiment only differentiated PTX and normal lungs in individual pigs and not between different animals. A larger validation study needs to be done to further evaluate the diagnostic accuracy of the microwave detector.

## Conflict of interest

The authors state no conflicts of interest.

## Institution

This preliminary study was conducted at Sandnes Education and Research Center Høyland (SEARCH), Sandnes, Norway.

